# Valedictory Address Delivered to the Graduating Class of Belleview Medical College, March 2, 1871

**Published:** 1872-01

**Authors:** Oliver Wendell Holmes

**Affiliations:** Parkman Professor of Anatomy and Physiology in the Medical School of Harvard University


### Selections.

### VALEDICTORY ADDRESS, DELIVERED TO THE GRADUATING CLASS OF BELLEVIEW MEDICAL COLLEGE, MARCH 2, 1871.

BY OLIVER WENDELL HOLMES, M. D.

Parkman Professor of Anatomy and Physiology in the Medical School of Harvard University.

* * * Yet pause a moment before you infer that your teachers must have been in fault when they furnished you with mental stores not directly convertible to practical purposes, and likely in a few years to lose their place in your memory. All systematic knowledge involves much that is not practical, yet it is the only kind of knowledge which satisfies the mind, and systematic study proves, in the long run, the easiest way of acquiring and retaining facts which are practical. There are many things which we can afford to forget, which yet it was well to learn. Your mental condition is not the same as if you had never known what you now in vain try to recall. There is a perpetual metempsychosis of thought, and the knowledge of to-day finds a soil in the forgotten facts of yesterday. You can not see anything new in the season of the guano you placed last year about the roots of your climbing plants, but it is blushing and breathing fragrance in your trellised roses; it has scaled your porch in the bee-haunted honey-suckle; it has found its way where the ivy is green; it is gone where the woodbine expands its luxuriant foliage. * * *

Your present plethora of acquirements will soon cure itself. Knowledge that is not wanted dies out like the eyes of the fishes of the Mammoth Cave. When you come to handle life and death as your daily business, your memory will of itself bid good-by to such inmates as the well-known foramina of the sphenoid bone and the familiar oxides of methylethyl-amyl-phenyl-ammonium. Be thankful that you have once known them, and remember that even the learned ignorance of a nomenclature is something to have mastered, and may furnish pegs to hang facts upon which would otherwise have strewed the floor of memory in disorder. * *

A certain amount of natural ability is requisite to make you a good physician, but by no means that disproportionate development of some special faculty which goes by the name of genius. A just balance of the mental powers is a good deal more likely to be useful than any single talent, even were it the power of observation, in excess. For a mere observer is liable to be too fond of facts for their own sake, so that, if he told the real truth, he would confess that he takes more pleasure in a *post mortem* examination which shows him what was the matter with a patient, than in a case which insists on getting well and leaving him in the dark as to its nature. Far more likely to interfere with the sound practical balance of mind is that speculative, theoretical tendency which has made so many men noted in their day, whose fame has passed away with their dissolving theories.

I warn you against all ambitious aspirations outside of your profession. Medicine is the most difficult of sciences and the most laborious of arts. It will task all your powers of body and mind if you are faithful to it. Do not dabble in the muddy sewer of politics, nor linger by the enchanted streams of literature, nor dig in far-off fields for the hidden waters of alien sciences. The great practitioners are generally those who concentrate all their powers on their business. If there are here and there brilliant exceptions, it is only in virtue of extraordinary gifts, and industry to which very few are equal. * * *

The public is a very incompetent judge of your skill and knowledge, but it gives its confidence most readily to those who stand well with their professional brethren, whom they call upon when they themselves or their families are sick, whom they choose to honorable offices, whose writings and teachings they hold in esteem. A man may be much valued by the profession and yet have defects which prevent his becoming a favorite practitioner, but no popularity can be depended upon as permanent which is not sanctioned by the judgment of professional experts, and with these you will always stand on your substantial merits. * * *

If there happened to be among my audience any person who wished to know on what principles the patient should choose his physician, I should give him these few precepts to think over:

Choose a man who is personally agreeable; for a daily visit from an intelligent, amiable, pleasant, sympathetic person will cost you no more than one from a sloven or a boor, and his presence will do more for you than any prescription the other will order.

Let him be a man of recognized good sense in other matters, and the chance is that he will be sensible as a practitioner.

Let him be a man who stands well with his professional brethren, whom they approve as honest, able, courteous.

Let him be one whose patients are willing to die in his hands, not one whom they go to for trifles and leave as soon as they are in danger, and who can say, therefore, that he never loses a patient.

